# Sex differences in the association of social network satisfaction and the risk for type 2 diabetes

**DOI:** 10.1186/s12889-017-4323-7

**Published:** 2017-05-02

**Authors:** K. Lukaschek, J. Baumert, J. Kruse, C. Meisinger, K.H. Ladwig

**Affiliations:** 1 Helmholtz Zentrum München, German Research Center for Environmental Health, Institute of Epidemiology II, Mental Health Research Unit, Ingolstaedter Landstr. 1, 85764 Neuherberg, Germany; 2 German Center for Diabetes Research (DZD), Munich, Germany; 3 Department of Psychosomatic Medicine and Psychotherapy, University of Gießen, Gießen, Germany; 4 Department of Psychosomatic Medicine and Psychotherapy, University of Marburg, Marburg, Germany; 5 MONICA/KORA Myocardial Infarction Registry, Central Hospital of Augsburg, Augsburg, Germany; 6 Department of Psychosomatic Medicine and Psychotherapy, Klinikum rechts der Isar, Technische Universität München, Munich, Germany

**Keywords:** Incident type 2 diabetes, Loneliness, Social isolation, Social network satisfaction, Sex differences

## Abstract

**Background:**

The role of an individual’s social network satisfaction (SNS) in the association of social isolation or living alone and incident type 2 diabetes (T2D) is unclear. We assessed the association of SNS with incident T2D and analysed potential modifications of the SNS-T2D association by social isolation or living alone.

**Methods:**

The study population (*N* = 6839 aged 25–74 years without diabetes at baseline) derived from the prospective population-based MONICA/KORA study (1989–2009). Social network satisfaction was assessed by a single item. Cox regression was used to estimate hazard ratios (HR) for SNS separately in men and women.

**Results:**

In men with low SNS, risk for incident T2D increased significantly (HR: 2.15, 95% CI: 1.33–3.48, *p* value 0.002). After additional adjustments for social isolation or living alone, the risk for incident T2D was still significant, albeit less pronounced (HRs 1.85 or 2.05, *p* values 0.001 or 0.004). The interaction analysis showed an increased T2D risk effect for low SNS compared to high SNS in women living in a partnership (HR: 2.11, 95% CI: 1.00–4.44, *p* value for interaction: 0.047) and for moderate SNS compared to high SNS in socially connected women (1.56, 1.01–2.39, 0.010).

**Conclusions:**

Further research is needed to address the complexities of the perception of social relationships and social interactions, or interdependence, especially when another major public health issue such as T2D is concerned.

**Electronic supplementary material:**

The online version of this article (doi:10.1186/s12889-017-4323-7) contains supplementary material, which is available to authorized users.

## Background

There is a growing body of research indicating the health risks of social isolation and loneliness [1]. Isolated individuals are at higher risk of increased morbidity and mortality, hospitalisation or poor cognitive function compared to those who are more socially integrated [2, 3]. These findings relate to the effects of objective social network support, but also for perceived (subjective) loneliness [1, 4].

Sex differences in social networks and social support have been discussed in previous studies [5–8]. There is consensus that women have a greater number of close relationships than men [9, 10]. However, it is debated whether women [11] or men [9] have more extensive social networks. Furthermore, sex has an important influence on support-relevant social interactions, and can have important effects on seeking and giving of social support in personal relations: women provide more emotional support to both men and women, and they receive more help in return [12]. It has also been found that sex moderates the association of social support and e.g. physical health, life satisfaction or vulnerability [5].

A limited number of population-based studies has suggested sex-specific associations of incident type 2 diabetes mellitus (T2D) and social relationships, or the lack thereof: Increased risk for incident T2D was associated with poor structural social support [13] and living alone [14] in men, and with low emotional support in women under work stress [15] and living without a current partner in women above the age of 70 years [16]. Furthermore, in women with impaired glucose tolerance (IGT), living alone was a risk factor for progressing to T2D [17]. Moreover, in subjects suffering from T2D, social support was associated with better self-rated health [18] and identified as a significant factor in the successful adaption of life-style changes [19, 20].

However, it remains unclear whether an individual’s *satisfaction* with their social network (SNS) plays a role in the association of lacking social relationships and incident T2D. Some socially isolated individuals may easily adapt to their few social contacts or even be satisfied with it, whereas others may be dissatisfied with their social network despite a large number of social contacts [4]. This unsatisfactory situation might be experienced as a constant stress burden with devastating consequences for the individual’s health [21].

Until now, no study has analysed whether a lack of social relationships affects the association between an individual’s SNS and incident T2D. We hypothesize that the SNS-T2D association is modified by living alone or social isolation, two established measures for the lack of social relationships. Thus, we (i) assess the association of SNS with incident T2D and (ii) analyse potential modifications of the SNS-T2D association by social isolation or living alone.

Due to the disparity between the sexes regarding the associations of social integration with T2D described above, we a priori performed all analyses separately for men and women.

## Methods

### Study design

Data were derived from the population-based Monitoring of Trends and Determinants in Cardiovascular Disease Augsburg (MONICA) studies [22, 23]: Three independent cross-sectional surveys covering the region of Augsburg (southern Germany) were carried out in 1984/1985 (S1), 1989/1990 (S2) and 1994/1995 (S3). Altogether, 13,426 individuals aged 25–74 years participated in at least one of these surveys.

Incident cases of T2D were assessed within the framework of Cooperative Health Research in the Region of Augsburg (KORA) using follow-up questionnaires in 1987/1988, 1997/1998, 2002/2003 and 2009 [24].

Social network satisfaction (SNS) was assessed in S2 and S3 (*n* = 9404). Participants with prevalent T2D or with other types of diabetes at baseline (*n* = 465) were excluded from the analyses leading to a source population of 8939 participants. Furthermore, we excluded participants with missing information on SNS, living alone or social network index (SNI) (*n* = 1082), on any of the variables at baseline required for the main analyses (*n* = 1138) and on T2D status at follow-up (*n* = 411). This lead to a final study population of 6839 participants aged 25–74 years without prevalent diabetes at baseline (3569 men, 3270 women).

The study follows the STROBE guidelines for observational studies [25].

### Assessment of exposure: social network satisfaction, living alone, social isolation

Social network satisfaction (SNS) was assessed by a single item “Overall, how satisfied are you with your relationship to friends and relatives?” („very satisfied“, „more or less satisfied “and „not very satisfied“, labelled as „high“, “moderate” and “low” SNS in the present analyses).

Living alone was assessed by asking the marital status. Participants answering unmarried and living alone, married but living separated, divorced or widowed were defined as living alone, otherwise as not living alone (yes/no).

Social support was assessed using the Social Network Index (SNI) [26] and was dichotomized: individuals with a score of 1 or 2 were categorized as socially isolated, individuals with a score of 3 or 4 were categorized as socially connected [27]. The dichotomized score indicates poor structural support versus good structural support [13].

### Assessment of confounders

Baseline information on sociodemographic variables, smoking habits, alcohol consumption, physical activity level, and sleep complaints was obtained in standardized personal interviews conducted by trained medical staff. All participants underwent an extensive standardized medical examination (including the assessment of BMI, hypertension and dyslipidaemia). All measurement procedures are described elsewhere in detail [24, 28],

### Sociodemographic risk factors

Participants were classified to have a low educational status if they completed less than 12 years of schooling.

### Behavioural risk factors

Information on smoking habits (never, past only, occasional, or regular) was provided by the participants. Assessment of alcohol intake was based on questionnaire data regarding weekday and weekend consumption of beer, wine and spirits. Study participants were asked about leisure time physical activity during winter and summer; they were classified as physically active if they exercised on average ≥ 1 h/week throughout the year (summer and winter).

### Clinical risk factors

BMI was calculated as weight in kg divided by height in m2. Actual hypertension was defined as blood pressure values ≥140/90 mmHg and/or use of an antihypertensive medication. Dyslipidaemia was defined as a ratio of total cholesterol to high-density lipoprotein cholesterol ≥5.0.

### Psychosocial risk factors

Sleep complaints were items regarding difficulties initiating sleep and difficulties maintaining sleep and were adopted from the Uppsala Sleep Inventory [29]. Participants were coded as having sleep complaints if they stated to have at least one of these difficulties often. Depressed mood was assessed using the depression and exhaustion subscale (DEEX) from the von Zerssen checklist [30]. It combines eight items (fatigue, tiredness, irritability, loss of energy, difficulty concentrating, inner tension, nervousness, anxiety) ranging from 0 to 3, leading to a Likert-like scoring range of 0 to 24 [31]. Participants in the top tertile of the depressive symptom distribution stratified by sex were considered as suffering from depressed mood.

### Assessment of incident type 2 diabetes

Incident cases of T2D were assessed in the follow-up. Self-reported T2D and the date of diagnosis were validated by hospital records or contacting the participants’ treating physicians. The hospital records of those deceased during the follow-up period without a diagnosis of T2D at baseline were examined and their last treating physicians were contacted [24].

### Statistical analyses

All analyses were performed separately for men and women. For unadjusted analyses, the chi-squared test was used to assess the association between categorical variables. Crude incidence rates for T2D were calculated by the number of incident T2D cases observed during the follow-up period divided by the sum of follow-up years of each person (person-years method). Cox regression was used to estimate hazard ratios (HR) for SNS. Potential confounding variables were chosen before the analyses driven by theoretical reasons. The proportional hazard assumption was confirmed for each variable by parallel curves when plotting the log(−log(survival probability)) for log (survival time).

All models were adjusted for age and survey (model 1), age, survey and cardiometabolic risk factors (model 2), age, survey, cardiometabolic risk factors and psychological risk factors (model 3) and additionally for social isolation (model 4) or living alone (model 5). Additionally, we ran model 3 with social isolation or living alone as exposure instead of SNS and compared their estimates with model 4 or model 5 (which included SNS as well).

To assess potential modifications of the association of SNS and incident T2D by social isolation or living alone, the respective interaction terms were included in the Cox models described above.

As sensitivity analysis, we repeated the logistic regression by applying an inverse probability weighting (IPW) approach [32] using weights stratified for age group (10-year intervals), sex and survey to deal with missing information in the source population (*n* = 8939). By this weighting, the analyses are based on the age, sex and survey distribution of the source population reducing potential bias of missing information which was mainly driven by age and sex. Robust variance estimations appropriate to the weighting scheme were computed using the SAS procedure SURVEYLOGISTIC.

For all statistical analyses, a *p* value <0.05 was considered to be statistically significant. SAS Version 9.2 for Windows (SAS Institute, Cary, NC) was used for all statistical analyses.

## Results

### Descriptive analyses

The study population comprised of 3569 men and 3270 women; mean age (standard deviation) was 48.0 years (±14.0) in men and 46.6 years (±13.3) in women. Additional file 1: Table S1 provides an overview of the distribution of study characteristics in men and women.

Overall, low social network satisfaction was more pronounced in men than in women (*p* < 0.001); in men, unsatisfying SNS was reported by 3.7% (low SNS) and 52.6% (moderate SNS) compared to proportions of 2.9% and 44.6% in women.

The association of living alone and social isolation with SNS in men and women is shown in Table 1. A substantial proportion of participants reported high SNS, despite living alone (46.5%) or being socially isolated (37.0%).

**Table 1:** Association* of living alone and social isolation with social network satisfaction in men (*n* = 3569) and in women (*n* = 3270)

		Living in a partnership	Living alone	Socially connected	Socially isolated
	SNS	%	%	%	%
Men	high	44.7	39.2	53.8	31.6
	moderate	52.3	53.8	45.3	61.3
	low	2.9	7.1	0.9	7.0
Women	high	52.5	52.5	62.9	42.2
	moderate	44.9	43.5	36.5	52.6
	low	2.6	4.1	0.6	5.2
Total	high	48.3	46.5	57.9	37.0
	moderate	48.9	48.1	41.3	56.9
	low	2.8	5.4	0.8	6.1

*p values were <0.001 except for association of living alone and SNS in women with *p* value 0.094.

### Crude incidence rate by SNS

Over a mean follow-up time of 14.0 years (standard deviation 4.7), a total number of 551 incident T2D cases (men: 333, women: 218) were observed. Among men, crude incidence T2D rates of 6–7 (high or moderate SNS at baseline) and 11 (low SNS at baseline) per 1000 person-years were observed. Among women, crude incidence rates of 4–5 (high or moderate SNS) and 6.7 (low SNS) per 1000 person-years were observed.

### Cox regression – main analyses

In men, the Cox regression revealed a significant increase in incident T2D for participants with low SNS compared to high SNS in all models (Table 2), even in the most comprehensive model 3 adjusted for age, survey, cardiometabolic risk factors and depressed mood (HR: 2.15, 95% CI: 1.33–3.48, *p* value 0.002). After additional adjustments for social isolation or living alone there was still a significant – albeit less pronounced – increase in incident T2D for participants with low SNS compared to participants with high SNS (HRs 1.85 and 2.05, *p* values 0.014 and 0.004) (Table 2). In women, no significant associations were found.

**Table 2:** Cox regression: increase in incident T2D risk for participants with low or moderate SNS compared to high SNS, different models

Model	SNS	Men (*n* = 3569)	Women (*n* = 3270)
		HR (95% CI)	HR (95% CI)
Model 1	moderate	0.99 (0.79–1.23)	1.00 (0.76–1.31)
	low	1.93 (1.21–3.08)*‡*	1.49 (0.76–2.95)
Model 2	moderate	1.06 (0.85–1.33)	1.07 (0.81–1.40)
	low	2.41 (1.50–3.87)*‡*	1.33 (0.67–2.64)
Model 3	moderate	1.00 (0.80–1.26)	1.04 (0.78–1.37)
	low	2.15 (1.33–3.48)*‡*	1.24 (0.62–2.47)
Model 4	moderate	0.94 (0.75–1.19)	1.02 (0.77–1.36)
	low	1.85 (1.13–3.03)*‡*	1.21 (0.60–2.43)
Model 5	moderate	0.98 (0.78–1.24)	1.04 (0.78–1.37)
	low	2.05 (1.27–3.32)*‡*	1.22 (0.61–2.43)

*‡ P* values were <0.05

Model 1: adjusted for age and survey

Model 2: adjusted additionally for cardiometabolic risk factors (smoking, alcohol consumption, physical inactivity, obesity, hypertension, dyslipidaemia)

Model 3: adjusted additionally for psychological risk factors (sleeping complaints, depressed mood)

Model 4: model 3 + additionally adjusted for social isolation

Model 5: model 3 + additionally adjusted for living alone

When running the model with social isolation or living alone as exposure instead of SNS, we found that the association of social isolation or living alone and incident T2D was only marginally confounded by low SNS: for social isolation, the HRs were 1.42 (95% CI: 1.14–1.77, *p* value 0.002) in a model without SNS and 1.39 (95% CI: 1.11–1.74, *p* value 0.004) with additional adjustment for SNS. Regarding living alone the respective estimates were 1.62 (95% CI: 1.22–2.15, *p* value 0.001) vs. 1.59 (95% CI: 1.20–2.12, *p* value 0.001).

### Cox regression – interaction analyses

In men, no significant modification of the SNS-T2D risk association by social isolation or living alone was estimated (data not shown).

In women, however, the interaction analysis showed an increased T2D risk for low SNS compared to high SNS in women living in a partnership in all models (Table 3), even in the fully adjusted model 3 (HR: 2.11, 95% CI: 1.00–4.44, *p* value for interaction: 0.047). Furthermore, the interaction analysis showed an increased T2D risk effect for moderate SNS compared to high SNS in women reporting being socially connected (Table 3; fully adjusted model 3: HR: 1.56, 95% CI: 1.01–2.39, *p* value for interaction 0.010).

**Table 3:** Impact of social network satisfaction on incident T2D risk by living alone or social isolation, estimated by Cox regression with different adjustments (interaction analyses), in women (*n* = 3270)

		Living in a partnership	Living alone	Socially connected	Socially isolated
Model	SNS	HR (95% CI) *p* value	HR (95% CI) *p* value	HR (95% CI) *p* value	HR (95% CI) *p* value
Model 1	moderate	1.13 (0.82–1.56) 0.465	0.75 (0.45–1.25) 0.274	**1.60** **(1.06–2.42)** **0.025**	0.70 (0.48–1.00) 0.051
	low	**2.41** **(1.16–5.01)** **0.018**	0.35 (0.05–2.55) 0.301	N/A^*^	1.33 (0.66–2.68) 0.424
*p* for interaction		0.191 (moderate), 0.075 (low)		0.003 (moderate)	
Model 2	moderate	1.20 (0.86–1.67) 0.278	0.80 (0.48–1.34) 0.403	**1.57** **(1.03–2.37)** **0.034**	0.77 (0.53–1.12) 0.168
	low	**2.22** **(1.06–4.64)** **0.034**	0.28 (0.04–2.07) 0.212	N/A^*^	1.25 (0.62–2.53) 0.538
*p* for interaction		0.194 (moderate), 0.046 (low)		0.007 (moderate)	
Model 3	moderate	1.17 (0.84–1.63) 0.355	0.76 (0.44–1.29) 0.308	**1.56** **(1.01–2.39)** **0.044**	0.74 (0.51–1.08) 0.120
	low	**2.11** **(1.00–4.44)** **0.049**	0.27 (0.04–1.98) 0.196	N/A^*^	1.17 (0.57–2.39) 0.675
*p* for interaction		0.186 (moderate) 0.047 (low)		0.010 (moderate)	

^*^estimates not possible due to low case numbers

Reference category: High social network satisfaction

#### Sensitivity analysis

We repeated the logistic regression by applying an inverse probability weighting (IPW) approach [32] using weights stratified for age group (10-year intervals), sex and survey to deal with missing information in the source population (*n* = 8939). These weighted analyses revealed comparable findings; HRs for high SNS versus low SNS in model 3 were 2.17 (95% CI 1.40–3.36, *p* value 0.001) in men and 1.23 (95% CI 0.66–2.31, *p* value 0.517) in women. Further adjustment for social isolation (model 4) and living alone (model 5) gave similar estimates as displayed in Table 2 (HRs 1.86 and 2.06, *p* values <0.01). Significance remained in the interaction analyses using this IPW approach (p for interaction regarding SNS were 0.018 instead of 0.010 in the unweighted analyses and regarding living alone 0.036 instead of 0.047, all in model 3).

## Discussion

### Findings of the main analyses

Overall, low SNS was associated with an increased risk for incident T2D in community-dwelling, middle-aged men. Adjustment for known cardiometabolic (smoking, alcohol consumption, physical inactivity, obesity, hypertension, dyslipidaemia) and psychological risk factors (sleeping complaints, depressed mood) further strengthened this association. Additional adjustment for social isolation or living alone attenuated the association of low SNS and incident T2D, but significance remained. Apparently, the unfavourable perception of one’s social network (as indicated by the low SNS) leads to a sustained stress burden. Previous studies have shown that chronic stress significantly contributes to an increased T2D risk [21, 33, 34]. We assume that the more public spheres men prefer for social contacts do not serve as adequately as confidants as the more multifaceted networks preferred by women [35].

Interestingly, the association of social isolation or living alone on incident T2D remained stable if low SNS was taken into account, indicating a low confounding of these effects by SNS.

### Findings of the interaction analyses

In men, no significant modification of the effect of low SNS on incident T2D risk was found, i.e. this association remained stable no matter whether the participant was socially isolated or not, or lived with a partner or not.

In contrast, the interaction analysis showed that in women the association of SNS and incident T2D was modified by social isolation and living alone: In women living in a partnership or being socially connected, the risk for incident T2D increased when satisfaction with the social network was low or moderate. The high emotional and psychological costs of maintaining a partnership or social network, despite the individual’s dissatisfaction with either of it, may lead to a sustained chronic stress situation and depression, possibly contributing to incident T2D. The underlying reason for this effect modification might be that the *quality* of the social network plays a very important role for women [36, 37] and that women generally have more and closer contact with their social network than men [38]. Social networks of family and friends are a supposed to be a source of support and associated with higher life-satisfaction [39]. However, they can also be associated with a greater level of stress, higher exposure to disputes or even lower self-esteem [40]. Thus, we argue that not only the size of a network, but also its quality may play an important role in the physical and mental health of an individual. The presence of a partner may not outweigh the absence of a satisfying social network, especially if there are conflicts with the partner. Previous research has shown that women who “self-silenced” during conflict had a four times higher mortality risk than women who did not self-silence [41]. Self-silencing is defined as the tendency to silence one’s thoughts and feelings to maintain safe relationships. Self-silencing thoughts and feelings can precipitate an overall self-negation through progressive devaluation of one’s own thought and beliefs [41] and thus, contribute severely to a constant stress-burden.

### Implications

Asking diabetic patients about living alone may be a useful starting point for understanding an individual’s social support [42]. However, this question alone does not offer a complete picture of an individual’s social relationships and the satisfaction with them [43]: SNS is an independent stress factor and thus, the patients’ satisfaction with their social network should be taken into account. The challenge for health professionals might be to find out whether a diabetic patient with very few social contacts is satisfied with this situation – has even chosen it – or not, and thus, envision different approaches to diabetes care and management. Clinicians and researchers need to ask more specific and detailed questions about individuals’ actual and perceived social support in order to identify subjects at highest risk for adverse health outcomes. Asking solely about chronic diseases or solely about living alone is bound to miss important and independent risk factors for adverse outcomes.

### Limitations and strengths

The strengths of the study are the population-based prospective design, the large sample size, and the long follow-up. Quality control in the MONICA/KORA studies was assured by extensive operation manuals, training and certification of interview and examination personnel, a pilot study well in advance of the main study, and an external quality assurance audit. Internal quality control was performed to regularly monitor all relevant aspects of data acquisition. All assessments were standardized. As limitation, all characteristics including social isolation were measured at baseline only. Moreover, SNS was assessed by a single item only. Furthermore, we cannot exclude reverse causality, i.e. that participants suffering from T2D report increased social isolation because their health condition limits their social contacts. Although the assessment of diabetic status was meticulously carried out, some of the non-diabetic participants might in fact have undetected diabetes. Although we controlled for the main diabetes-related risk factors, we cannot exclude some unmeasured or residual confounding (e.g. cultural and/or immigration background, or health and social inequalities).

## Conclusions

Low social network satisfaction is an independent predictor of type 2 diabetes in men. In women, the presence of a partner may not outweigh the absence of a satisfying social network. Thus, specific attention should be given to women who are dissatisfied with their social network. Further research is needed to address the complexities of the perception of social integration, social interactions, or interdependence, especially when another major public health issue such as T2D comes into the picture.

## Additional file

Additional file 1: Table S1. Description of study population. (DOCX 18 kb)

## Abbreviations

HDL: High density lipoprotein
HR: Hazard ratio
IGT: Impaired glucose tolerance
MONICA/KORA: Monitoring of trends and determinants in CArdiovascular disease/Cooperative Health Research in the Augsburg Region
S1, S2, S3: Survey 1, 2,3
SD: Standard deviation
SNI: Social network index
SNS: Social network satisfaction
T2D: Type 2 Diabetes

## References

[1] Shankar A, McMunn A, Demakakos P, Hamer M, Steptoe A. Social Isolation and Loneliness: Prospective Associations With Functional Status in Older Adults. Health Psychol. 2017;36(2):179-87. 10.1037/hea0000437 27786518

[2] Holt-Lunstad J, Smith TB, Layton JB (2010). Social relationships and mortality risk: a meta-analytic review. PLoS Med.

[3] Shankar A, McMunn A, Banks J, Steptoe A (2011). Loneliness, social isolation, and behavioral and biological health indicators in older adults. Health Psychol.

[4] Holt-Lunstad J, Smith TB, Baker M, Harris T, Stephenson D (2015). Loneliness and social isolation as risk factors for mortality: a meta-analytic review. Perspect Psychol Sci.

[5] Matud MP, Ibanez I, Bethencourt JM, Marrero R, Carballeira M (2003). Structural gender differences in perceived social support. Personal Individ Differ.

[6] Nicklett EJ: Sex, Health Behaviors and Social Support: Functional Decline among Older Diabetics. Am Med J. 2012: 3(2). 10.3844/amjsp.2012.82.92 PMC3866132 24358419

[7] Stokes JP, Wilson DG (1984). The inventory of socially supportive behaviors: dimensionality, prediction, and gender differences. Am J Community Psychol.

[8] Verbrugge LM, Wingard DL (1987). Sex differentials in health and mortality. Women Health.

[9] Fuhrer R, Stansfeld SA, Chemali J, Shipley MJ (1999). Gender, social relations and mental health: prospective findings from an occupational cohort (Whitehall II study). Soc Sci Med.

[10] Knoll N, Schwarzer R, Weidner G, Kopp M, Kristenson M (2002). Gender and Age Differences in Social Support: A Study of East German Migrants. Heart disease: Environment, stress, and gender.

[11] McFarlane AH, Neale KA, Norman GR, Roy RG, Streiner DL (1981). Methodological issues in developing a scale to measure social support. Schizophr Bull.

[12] Kessler RC, McLeod JD, Wethington E, Sarason IG, Sarason BR (1985). The costs of caring: A perspective on the relationship between sex and psychological distress. Social Support: Theory, research and applications.

[13] Altevers J, Lukaschek K, Baumert J, Kruse J, Meisinger C, Emeny RT, Ladwig KH (2016). Poor structural social support is associated with an increased risk of Type 2 diabetes mellitus: findings from the MONICA/KORA Augsburg cohort study. Diabet Med.

[14] Meisinger C, Kandler U, Ladwig KH (2009). Living alone is associated with an increased risk of type 2 diabetes mellitus in men but not women from the general population: the MONICA/KORA Augsburg Cohort Study. Psychosom Med.

[15] Norberg M, Stenlund H, Lindahl B, Andersson C, Eriksson JW, Weinehall L (2007). Work stress and low emotional support is associated with increased risk of future type 2 diabetes in women. Diabetes Res Clin Pract.

[16] Strodl E, Kenardy J (2006). Psychosocial and non-psychosocial risk factors for the new diagnosis of diabetes in elderly women. Diabetes Res Clin Pract.

[17] Lidfeldt J, Nerbrand C, Samsioe G, Agardh CD (2005). Women living alone have an increased risk to develop diabetes, which is explained mainly by lifestyle factors. Diabetes Care.

[18] Eller M, Holle R, Landgraf R, Mielck A (2008). Social network effect on self-rated health in type 2 diabetic patients--results from a longitudinal population-based study. Int J Public Health.

[19] Ashida S, Heaney CA (2008). Differential associations of social support and social connectedness with structural features of social networks and the health status of older adults. J Aging Health.

[20] Crotty MM, Henderson J, Ward PR, Fuller J, Rogers A, Kralik D, Gregory S (2015). Analysis of social networks supporting the self-management of type 2 diabetes for people with mental illness. BMC Health Serv Res.

[21] McEwen BS, Stellar E (1993). Stress and the individual. Mechanisms leading to disease. Arch Intern Med.

[22] Holle R, Happich M, Lowel H, Wichmann HE, Group MKS (2005). KORA--a research platform for population based health research. Gesundheitswesen.

[23] Lowel H, Doring A, Schneider A, Heier M, Thorand B, Meisinger C, Group MKS (2005). The MONICA Augsburg surveys--basis for prospective cohort studies. Gesundheitswesen.

[24] Meisinger C, Thorand B, Schneider A, Stieber J, Döring A, Löwel H (2002). Sex differences in risk factors for incident type 2 diabetes mellitus: the MONICA Augsburg cohort study. Arch Intern Med.

[25] von Elm E, Altman DG, Egger M, Pocock SJ, Gotzsche PC, Vandenbroucke JP, Initiative S (2007). The Strengthening the Reporting of Observational Studies in Epidemiology (STROBE) statement: guidelines for reporting observational studies. Lancet.

[26] Berkman LF, Syme SL (1979). Social networks, host resistance, and mortality: a nine-year follow-up study of Alameda County residents. Am J Epidemiol.

[27] Pantell M, Rehkopf D, Jutte D, Syme SL, Balmes J, Adler N (2013). Social isolation: a predictor of mortality comparable to traditional clinical risk factors. Am J Public Health.

[28] Evans A, Tolonen H, Hense HW, Ferrario M, Sans S, Kuulasmaa K, Project WM (2001). Trends in coronary risk factors in the WHO MONICA project. Int J Epidemiol.

[29] Mallon L, Broman JE, Hetta J. Sleep complaints predict coronary artery disease mortality in males: a 12-year follow-up study of a middle-aged Swedish population. J Intern Med. 2002;251(3):207–16. 10.1046/j.1365-2796.2002.00941.x 11886479

[30] Zerssen D (1976). Die Beschwerden-Liste Klinische Selbstbeurteilungsfrageboegen aus dem Muenchener Psychiatrischen Informationssystem (Psychis).

[31] Ladwig KH, Marten-Mittag B, Baumert J, Lowel H, Doring A, Investigators K (2004). Case-finding for depressive and exhausted mood in the general population: reliability and validity of a symptom-driven diagnostic scale. Results from the prospective MONICA/KORA Augsburg Study. Ann Epidemiol.

[32] Seaman SR, White IR (2013). Review of inverse probability weighting for dealing with missing data. Stat Methods Med Res.

[33] Eriksson AK, Ekbom A, Granath F, Hilding A, Efendic S, Ostenson CG (2008). Psychological distress and risk of pre-diabetes and Type 2 diabetes in a prospective study of Swedish middle-aged men and women. Diabet Med.

[34] Novak M, Bjorck L, Giang KW, Heden-Stahl C, Wilhelmsen L, Rosengren A (2013). Perceived stress and incidence of Type 2 diabetes: a 35-year follow-up study of middle-aged Swedish men. Diabet Med.

[35] Zebhauser A, Hofmann-Xu L, Baumert J, Hafner S, Lacruz ME, Emeny RT, Doring A, Grill E, Huber D, Peters A (2014). How much does it hurt to be lonely? Mental and physical differences between older men and women in the KORA-Age Study. Int J Geriatr Psychiatry.

[36] Due P, Holstein B, Lund R, Modvig J, Avlund K (1999). Social relations: network, support and relational strain. Soc Sci Med.

[37] Josselson R (1987). Finding Herself. Pathways to Identity Development in Women.

[38] Hempler NF, Joensen LE, Willaing I (2016). Relationship between social network, social support and health behaviour in people with type 1 and type 2 diabetes: cross-sectional studies. BMC Public Health.

[39] Tomini F, Tomini SM, Groot W (2016). Understanding the value of social networks in life satisfaction of elderly people: a comparative study of 16 European countries using SHARE data. BMC Geriatr.

[40] Kawachi I, Berkman LF (2001). Social ties and mental health. J Urban Health.

[41] Eaker ED, Sullivan LM, Kelly-Hayes M, D'Agostino RB, Benjamin EJ (2007). Marital status, marital strain, and risk of coronary heart disease or total mortality: the Framingham Offspring Study. Psychosom Med.

[42] Perissinotto CM, Covinsky KE (2014). Living alone, socially isolated or lonely--what are we measuring?. J Gen Intern Med.

[43] Ennis SK, Larson EB, Grothaus L, Helfrich CD, Balch S, Phelan EA (2014). Association of living alone and hospitalization among community-dwelling elders with and without dementia. J Gen Intern Med.

