# Supplementary material for: Sex differences in the association of social network satisfaction and the risk for type 2 diabetes
Source: BMC Public Health. 2017 May 2;17:379. doi: 10.1186/s12889-017-4323-7 (PMC5414370; doi:10.1186/s12889-017-4323-7)
Supplement: Additional file 1: Table S1. — Description of study population. (DOCX 18 kb) [file 12889_2017_4323_MOESM1_ESM.docx]

**Supplementary Table 1: Description of study population**

|  | **Men (n=3,569)** | **Women (n=3,270)** |
| --- | --- | --- |
| Age (mean,SD), years | 48.0 (14.0) | 46.6 (13.3) |
| Low educational level | \| 61.1 \| \| --- \| | \| 76.9 \| \| --- \| |
| History of parental diabetes |  |  |
| No | \| 62.2 \| \| --- \| | \| 63.8 \| \| --- \| |
| Yes | \| 17.4 \| \| --- \| | \| 20.6 \| \| --- \| |
| Unknown | \| 20.5 \| \| --- \| | \| 15.6 \| \| --- \| |
| Current smoker | \| 30.1 \| \| --- \| | \| 22.5 \| \| --- \| |
| Alcohol consumption |  |  |
| No | \| 17.8 \| \| --- \| | \| 42.2 \| \| --- \| |
| Moderate | \| 53.1 \| \| --- \| | \| 39.5 \| \| --- \| |
| High | \| 29.1 \| \| --- \| | \| 18.4 \| \| --- \| |
| Physical inactivity | \| 52.8 \| \| --- \| | \| 57.9 \| \| --- \| |
| Obesity | \| 17.4 \| \| --- \| | \| 17.5 \| \| --- \| |
| Actual hypertension | \| 44.0 \| \| --- \| | \| 28.9 \| \| --- \| |
| TC/HDL-C > 5 | \| 45.3 \| \| --- \| | \| 15.8 \| \| --- \| |
| Sleeping disturbances | \| 18.1 \| \| --- \| | \| 23.2 \| \| --- \| |
| Depressed mood | \| 37.7 \| \| --- \| | \| 35.85 \| \| --- \| \|  \| |
| Incident T2D |  |  |
| N / % | \| 333 / 9.3 \| \| --- \| | \| 218 / 6.7 \| \| --- \| |
| FU time (mean,SD) | \| 13.7 (5.0) \|  \| \| --- \| --- \| | 14.6 (4.3) |
| Incidence rate/1,000 pyrs | 6.8 | 4.6 |
